# MiRNA-223-5p inhibits hypoxia-induced apoptosis of BMSCs and promotes repair in Legg-Calvé-Perthes disease by targeting CHAC2 and activating the Wnt/β-catenin signaling pathway

**DOI:** 10.1371/journal.pone.0315230

**Published:** 2025-01-24

**Authors:** Jiafei Yang, Tianjiu Zhang, Xingtao Zhu, Zhexi He, Xu Jiang, Song Yu, Huajian Gu

**Affiliations:** 1 School of Clinical Medicine, Guizhou Medical University, Guiyang, China; 2 Department of Pediatric Surgery, The Affiliated Hospital of Guizhou Medical University, Guizhou Medical University, Guiyang, China; 3 Department of Pediatric Surgery, The Affiliated Hospital of Zunyi Medical University, Zunyi Medical University, Zunyi, China; 4 Guizhou University of Traditional Chinese Medicine, Guiyang, China; 5 Zunyi Medical And Pharmaceutical College, Zunyi, China; University of Vermont, UNITED STATES OF AMERICA

## Abstract

Legg-Calvé-Perthes disease (LCPD) involves femoral head osteonecrosis caused by disrupted blood supply, leading to joint deformity and early osteoarthritis. This study investigates the role of miRNA-223-5p in regulating hypoxia-induced apoptosis and enhancing osteogenesis in bone marrow mesenchymal stem cells (BMSCs). Utilizing a juvenile New Zealand white rabbit model of LCPD established through femoral neck ligation, we transfected BMSCs with miR-223-5p mimics, inhibitors, and controls, followed by hypoxic exposure. The impact of miR-223-5p on BMSC apoptosis was assessed using qPCR, Western blotting, and dual-luciferase reporter assays, focusing on the Wnt/β-catenin signaling pathway. In vivo, we evaluated the effects of transplanting miR-223-5p-overexpressing BMSCs into the LCPD model. Our results indicate that miR-223-5p is downregulated under hypoxic conditions. Overexpression of miR-223-5p in BMSCs inhibited hypoxia-induced apoptosis and activated the Wnt/β-catenin pathway by directly targeting CHAC2. In vivo, miR-223-5p-overexpressing BMSCs enhanced femoral head osteogenesis and reduced necrosis in the LCPD model. These findings suggest that miR-223-5p inhibits hypoxia-induced apoptosis in BMSCs by targeting CHAC2 and activating the Wnt/β-catenin pathway, proposing miR-223-5p as a promising target for improving bone repair in ischemic conditions.

## 1. Introduction

Legg-Calvé-Perthes disease (LCPD) is an idiopathic osteonecrosis marked by the necrosis of the femoral head and cartilage due to an interruption of blood supply, leading to progressive deformity and degenerative osteoarthritis [1]. LCPD affects approximately 5 children per 1,000,000 aged 2 to 14 [2]. The healing process often results in various degrees of joint dysfunction and deformity, potentially progressing to early osteoarthritis [3]. Despite its rarity, LCPD has a profound impact on the quality of life of affected children, causing long-term pain and impaired mobility. Current treatment strategies are aimed at maintaining femoral head shape and preventing further damage, but the outcomes vary significantly depending on the stage at diagnosis and the age of the patient [4]. Moreover, the unpredictable course of the disease and the limitations of both conservative and surgical treatments often fail to restore full function or prevent progression to osteoarthritis [5]. Therefore, there is a pressing need to explore novel therapeutic approaches that can effectively promote femoral head repair and reduce necrosis, ultimately improving patient outcomes.

Angiographic studies by Atsumi have shown that occlusion of the lateral epiphyseal arteries occurs in children with LCPD [6]. This blockage results in osteonecrosis in the femoral head, characterized by reduced activity and apoptosis of osteoblasts and bone marrow mesenchymal stem cells (BMSCs) under hypoxic conditions. Research has demonstrated that local BMSC injections can promote bone repair, offering a novel treatment approach for early LCPD [7–9]. However, the hypoxic environment limits the osteogenic repair capacity of transfected cells [10]. Therefore, it is essential to investigate how femoral head necrosis causes apoptosis in transfected BMSCs to enhance the efficacy of BMSC transplantation in LCPD. Identifying new targets to inhibit hypoxia-induced apoptosis of BMSCs and improving the effectiveness of BMSC transplantation are vital steps in advancing LCPD treatment.

MicroRNAs (miRNAs) are noncoding RNA molecules, usually 18–25 nucleotides long, that bind to the 3’ untranslated region of target mRNAs, inhibiting translation and causing gene silencing [11, 12]. They regulate over 60% of human protein-coding genes, playing critical roles in physiological processes and cellular functions like differentiation and apoptosis [13]. For example, miRNA-10a-5p induces apoptosis in chicken myoblasts by targeting BCL6 (B-cell lymphoma 6) [14], while miRNA-210 promotes apoptosis in rat neurons during cerebral ischemia via the HIF-1α-VEGF pathway [15]. Additionally, miRNAs form competing endogenous RNA (ceRNA) networks with lncRNAs and mRNAs, such as LINC00958 acting as a ceRNA for miR-484, influencing mitochondrial function and apoptosis in granulosa cells under oxidative stress [16]. This study identified abnormal miRNAs in rabbits with LCPD using miRNA microarray assays. Previous research links miR-223-5p to alkaline phosphatase (ALP) activity and anti-apoptotic functions [17, 18]. However, its role in BMSC anti-apoptosis remains underexplored. Understanding miR-223-5p’s function could enhance BMSC transplantation efficacy for LCPD treatment.

Glutathione (GSH), a crucial tripeptide (γ-glutamyl-cysteinyl glycine), is essential for detoxification, redox signaling, cell proliferation, and apoptosis [19]. Recently, two isoforms, CHAC1 (CHAC cation transport regulator homolog 1) and CHAC2 (CHAC cation transport regulator homolog 2), have been identified as key regulators of GSH homeostasis in eukaryotes [20, 21]. CHAC1 significantly contributes to GSH degradation, with its overexpression leading to reduced GSH levels, elevated intracellular reactive oxygen species (ROS), and increased apoptosis [22, 23]. The role of CHAC2 in GSH degradation is more controversial. Some studies suggest that CHAC2 competes with CHAC1 to maintain GSH homeostasis and mitigate CHAC1-mediated GSH degradation, with CHAC2 overexpression increasing GSH levels and decreasing ROS [24, 25]. Conversely, other studies report that CHAC2 reduces GSH levels and raises ROS in lung adenocarcinoma cells [26]. Thus, the role of CHAC2 in apoptosis remains unclear and requires further investigation.

In this study, we investigated the effects and mechanisms of the interaction between miR-223-5p and CHAC2 on hypoxia-induced apoptosis of BMSCs. We also evaluated the potential of inhibiting hypoxia-induced apoptosis of BMSCs as a treatment strategy for early LCPD. Our findings contribute to identifying novel targets and developing methods to inhibit hypoxia-induced apoptosis in BMSCs, ultimately enhancing the efficacy of BMSC transplantation for treating LCPD.

In this study, we explored the role of miR-223-5p in preventing apoptosis within the context of LCPD, both in vitro and in vivo, with the aim of identifying a promising new target for early treatment.

## 2. Materials and methods

### 2.1 Animals

We established LCPD models using 2-month-old juvenile New Zealand white rabbits weighing 1.5 to 2.0 kg, obtained from the Laboratory Animal Center of Guizhou Medical University (Guiyang, China). The modeling was performed using a femoral neck ligation method on 2-month-old rabbits for a duration of 4 weeks. For untreated rabbits, sacrifice was performed 4 weeks after ligation, whereas for locally treated rabbits, ligation was followed by local injection treatment, and they were housed for an additional 4 weeks before sacrifice. The age of the experimental animals did not exceed 4 months upon model completion. The final age of the young rabbits aligns with the onset age of LCPD disease in humans. All experiments were approved by the Experimental Animal Ethics Committee of Guizhou Medical University (Grant No. 2201637). The rabbits were housed in a dry, ventilated environment at a controlled temperature of approximately 25°C, provided with a complete formula diet, and had normal access to water. Their environmental conditions were kept consistent.

All experimental procedures were conducted in accordance with the guidelines of the Institutional Animal Care and Use Committee of Laboratory Animals, as published by the US National Institutes of Health (NIH Publication No. 85–23, revised in 1996).

### 2.2 Animal model and grouping

In our study, we used a randomized allocation method to assign subjects to different groups. To minimize bias and ensure data integrity, investigators who handled the animals and measured the endpoints were blinded to the group assignments. This blinding was maintained throughout the study to ensure objective handling and assessment of the animals.

The rabbit was positioned laterally after being anesthetized with 30 mg/kg sodium pentobarbital (Sigma-Aldrich, USA) administered via an ear vein. The surgical area was disinfected and draped. A 2-cm incision was made, extending from 1 cm above the greater trochanter to the mid-femur on the left side. Blunt dissection of the tensor fascia and gluteus maximus muscle was followed by extreme flexion and internal rotation of the hip to expose the joint capsule. The femoral head was dislocated, and the Ligamentum teres was cut, severing the blood supply. Using a curved clamp, non-absorbable sutures were placed around the femoral neck, severing the vascular supply. The hip was then reduced, and the wound was sutured.

**Fig 1:**
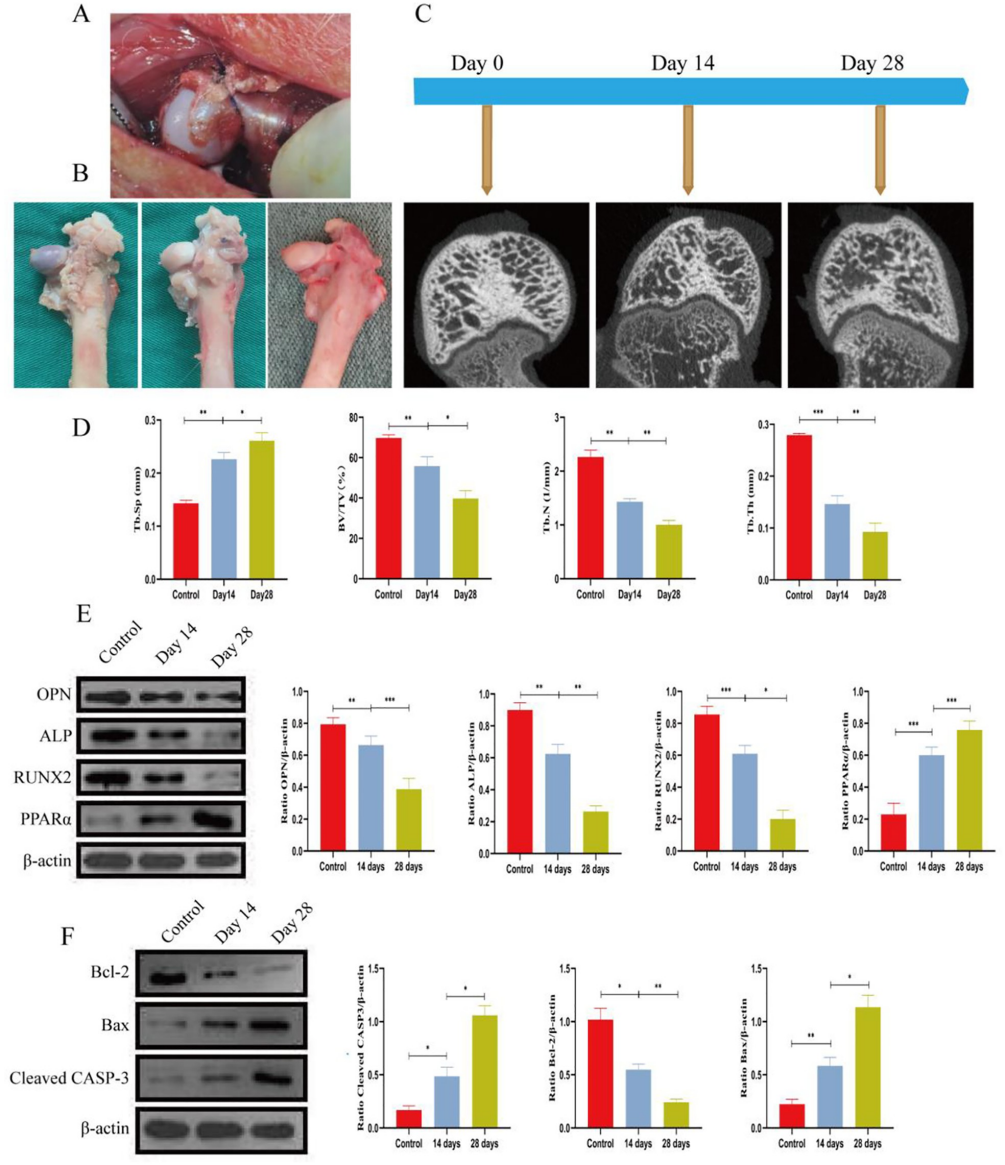
The rabbit femoral neck cerclage model leads to local apoptosis in the rabbit femoral head. Micro-CT and Western blot (WB) analyses were conducted to assess structural changes in the femoral head and alterations in bone metabolism markers following femoral neck ligation in juvenile rabbits. Rabbits were sacrificed at 14 and 28 days post-ligation, and femoral head tissues were harvested for subsequent micro-CT imaging and WB analysis. **(A)** Schematic diagram of cerclage on the femoral neck of a young rabbit. **(B)** Macroscopic morphological changes in the control group and surgery-induced group (from left to right: 0 days, 14 days, and 28 days). **(C)** Micro-CT monitoring performed before and after surgery. **(D)** Quantitative analysis of trabecular number, trabecular thickness, and volume fraction of new bone tissue. **(E, F)** Expression levels of Runx2, OPN, ALP, PPARα, Bcl-2, Bax, and CASP-3 detected by Western blotting. Data are shown as the means ± SD. *p<0.05, **p<0.01, ***p<0.01 (n = 3).

The second stage of the operation was performed 4 weeks later using the same anesthesia protocol. Under fluoroscopic guidance, drilling was done, and mesenchymal stem cells were locally injected. The model establishment process is shown in Fig 1A. A total of 1 × 10^6 lentiviral-transfected miR-223-5p-overexpressing BMSCs or NC-BMSCs were locally injected into the femoral head drilling site of the experimental group or NC group at the 4-week point of model establishment. The control group rabbits were given only saline.

### 2.3 Cell isolation and culture

BMSCs were isolated from the femurs and tibias of young male New Zealand white suckling rabbits weighing 100 to 150 g. To culture BMSCs, bone marrow nucleated cells (BMNCs) were resuspended in Dulbecco’s Modified Eagle Medium (DMEM; Gibco, USA) supplemented with 10% fetal bovine serum (FBS; Gibco, USA) and 1% penicillin/streptomycin (HyClone). The cells were seeded into 25 cm^2^ culture flasks and incubated at 37°C in a humidified atmosphere with 5% CO2. After 3 days, nonadherent cells were removed by replacing the culture medium. When the adherent cells reached 80–90% confluence, they were trypsinized with 0.25% trypsin (Gibco) and subcultured at a density of 1 × 10^4^ cells/cm^2^. BMSCs at passages three to four were used for subsequent experiments.

BMSCs at passages three or four were transfected with miR-223-5p mimics, mimic negative controls (NC), inhibitors, and inhibitor NC using Lipofectamine 3000 Reagent (Invitrogen). BMSCs exposed to hypoxic conditions were designated as the model group, while those kept under normoxic conditions were designated as the control group.

### 2.4 Osteogenic differentiation of BMSCs

Second-generation BMSCs were seeded in six-well plates. Once the cells reached approximately 60% confluence in the culture flasks, the experimental group received osteogenic induction medium in accordance with the instructions from the BMSC Osteogenic Induction Kit. The control group, however, was maintained in complete L-DMEM. After 14 days of induction, the cells were fixed, and osteogenic differentiation was evaluated. Alkaline phosphatase (ALP) activity was assessed through ALP staining, and calcium deposits were identified using 0.1% alizarin red staining.

### 2.5 Lipogenic differentiation of BMSCs

Second-generation BMSCs were plated in six-well plates. Upon reaching full confluence or slight over confluence, the medium for the experimental group was switched to adipogenic differentiation medium, following the protocol provided in the BMSC Adipogenic Induction Kit. In contrast, the control group continued in complete L-DMEM. After 21 days of induction, lipid droplets were visualized using oil red O staining.

### 2.6 Chondrogenic differentiation of BMSCs

Second-generation BMSCs were cultured in six-well plates. When the cells reached approximately 60% confluence, the experimental group’s medium was substituted with chondrogenic induction medium as outlined in the BMSC Chondrogenic Induction Kit (Cyagen Biosciences, Santa Clara, CA, USA). Meanwhile, the control group was maintained in complete L-DMEM. Following a four-week induction period, cartilage acidic mucopolysaccharides were detected through Alcian blue staining (Cyagen Biosciences).

### 2.7 Identification of surface antigens on BMSCs

Second-generation BMSCs were prepared at a density of 2 × 10^7^ cells/mL. In the control group, 50 μL of buffer was added, while the single-label group received 5 μL of one antibody per tube, including CD34, CD45, CD90, CD90, CD105 followed by 45 μL of buffer. For the multicolor group, 5 μL of each antibody was added to a single tube, along with 25 μL of buffer. Subsequently, 50 μL of cell suspension was added to each tube. The samples were incubated at room temperature for 30 minutes, washed twice with staining buffer, and resuspended in 500 μL of buffer. Flow cytometry analysis was performed using a system from Beckman Coulter Life Sciences.

### 2.8 Cell hypoxia model

To induce hypoxia, third- or fourth-passage BMSCs were continuously exposed to a gas mixture of 0% oxygen, 95% nitrogen, and 5% carbon dioxide for 48 hours.

### 2.9 Micro-CT scanning

To evaluate the bone morphology of the femoral heads in young rabbits, high-resolution micro-CT (Alteslar, SKYSCAN 1276, Bruker, Konitch, Belgium) was performed with scanning parameters of 25 μm, 55 kV, and 200 mA. Trabecular bone parameters, including bone mineral density (BMD, mg/cm^3^), bone volume (BV, mm^3^), bone volume per tissue volume (BV/TV, %), trabecular thickness (Tb.Th, mm), and trabecular number (Tb.N, 1/mm), were analyzed using CT Analyzer software (CTAN, Bruker).

### 2.10 Hematoxylin and eosin (H&E) staining

The femoral heads from young rabbits were fixed in 4% paraformaldehyde for 48 hours and decalcified in 10% diamine ethylene tetraacetic acid (EDTA, Sigma) for 8 weeks. The decalcified femoral heads were embedded in paraffin, sectioned into 7 μm thick slices, and mounted on slides. The slices were stained with H&E and sealed with neutral resin. The stained sections were examined under an AxioCam HRC camera attached to a microscope (Carl Zeiss, Oberkochen, Germany).

### 2.11 Real-time quantitative PCR

We extracted RNA from the femoral heads and BMSCs using column affinity purification (BioTeke, Beijing, China) and synthesized complementary DNAs (cDNAs) with M-MuLV RT Master Mix and Oligo(dT) (Sangon Biotech, Shanghai, China). Real-time PCR was conducted on a StepOnePlus system (Applied Biosystems, Foster City, CA, USA) in 96-well plates using specific primers and SYBR Green Mix (Sangon Biotech). The primer sequences are listed in Table 1.

**Table 1:** Sequences of primers used for real-time qPCR analysis.

Gene	Forward primer (5’-3’)	Reverse primer (5’-3’)
Ocu-miR-223-5p	5’-AACACGCCGTGTATTTGACAAG-3’	5’-GTCGTATCCAGTGCAGGGT
Ocu-miR-17-5P	5’-AACACGCCAAAGTGCTTACAG-3’	5’-GTCGTATCCAGTGCAGGGT-3’
Ocu-miR-363-3P	5’-AACACGCAATTGCACGGTAT-3’	5’-GTCGTATCCAGTGCAGGGT-3’
Ocu-miR-144-5P	5’-AGCCAGCGGGATATCATCATATA-3’	5’-GTCGTATCCAGTGCAGGGT-3’
Ocu-miR-187-3P	5’-AACAGTGTCGTGTCTTGTGTT-3’	5’-GTCGTATCCAGTGCAGGGT-3’
Ocu-miR-223-3P	5’-AACACGCTGTCAGTTTGTCAAA-3’	5’-GTCGTATCCAGTGCAGGGT-3’
Ocu-miR-365-3p	5’-AACACGCTAATGCCCCTAAAA-3’	5’-GTCGTATCCAGTGCAGGGT-3’
U6	5’-GCAAACTCGATCACTACCTCTGC-3’	5’-ACAAAGAACCACCTCAGTAGTGTC-3’

### 2.12 Western blotting

BMSCs were cultured in 6-well plates at a density of 3 × 10^5 cells per well. Proteins were extracted using radioimmunoprecipitation assay buffer (RIPA, Solarbio, Beijing, China). The supernatant was collected after centrifugation at 14,000 × g for 5 minutes at 4°C. Following determination and standardization of the total protein concentration in each group, samples with equal protein content were separated using 10% sodium dodecyl sulfate-polyacrylamide gel electrophoresis (SDS-PAGE; EpiZyme Biotech, Shanghai, China) and transferred to nitrocellulose membranes. The membranes were blocked with QuickBlock Blocking Buffer (EpiZyme Biotech) for 15 minutes to prevent nonspecific binding and then incubated overnight with primary antibodies at 4°C. The following day, the membranes were incubated with goat anti-rabbit/mouse IgG (H+L) HRP secondary antibodies for 1 hour. Finally, protein bands were visualized using an Enhanced ECL Chemiluminescent Substrate Kit (Invitrogen), and the relative gray values were analyzed with Image Lab 3.0 software (Bio-Rad, Hercules, CA, USA).

### 2.13 Dual-luciferase reporter assay

The CHAC2 3′UTR sequence containing either the wild-type (WT) or mutant (MUT) miR-223-5p putative binding region was amplified by RiboBio (Guangzhou, China) and inserted into the pGL3-GP73-3′UTR plasmid (Invitrogen). The plasmids and miR-223-5p mimics (or miR-NC) were co-transfected into cells using Lipofectamine 3000 (Invitrogen). After 48 hours of transfection, luciferase activity was measured with the Dual-Luciferase Reporter Assay System (Promega, USA).

### 2.14 Annexin V-fluorescein isothiocyanate (FITC) / propidium iodide (PI)

Third- or fourth-passage BMSCs were washed with PBS, after which 5 μL of Annexin V-FITC and 5 μL of PI were added following the instructions of the Annexin V-FITC apoptosis detection kit (Elabscience, Wuhan, China). The cells were gently vortexed, incubated at room temperature in the dark for 15 minutes, and then analyzed by flow cytometry.

### 2.15 TdT-mediated dUTP nick-end labeling (TUNEL) / 4′,6-diamidino-2-phenylindole (DAPI)

Third- or fourth-passage BMSCs were fixed with 4% paraformaldehyde (Solarbio, Beijing, China) at 4°C for 1–2 hours and permeabilized with 0.3% Triton X-100 (Coolaber) for 10 minutes. The TUNEL detection solution (Elabscience, Wuhan, China) was then added, and the samples were incubated at 37°C for 60 minutes in the dark. After incubation, the samples were washed with PBS and stained with DAPI for 5 minutes.

### 2.16 RNA interference and plasmid transfection

siRNA and overexpression plasmids were supplied by APExBIO. RNA interference and plasmid transfection protocols were followed according to the manufacturer’s instructions. In brief, siRNA/plasmid and GP-transfect-Mate were diluted and added to 6-well plates when BMSCs reached 70% confluency. The cells were incubated for 24 hours. Transfection efficacy was evaluated by measuring the relative gene and protein expression levels.

### 2.17 Lentiviral transfections

Lentiviruses were obtained from China Shanghai Genechem Co., Ltd., based on the optimal multiplicity of infection (MOI = 80) and transfection conditions established from preliminary experiments. Second-generation BMSCs were infected with these lentiviruses, with blank control and negative control groups also established. After 12 hours, the culture medium was replaced with complete L-DMEM. On the fourth day post-infection, a stable strain was selected by adding 2 μg/mL puromycin. Once all cells in the blank control group died, the puromycin concentration was lowered to 1 μg/mL to maintain the selection.

### 2.18 Microarray and bioinformatic analyses

The total RNA was extracted using TRIzol reagent (Invitrogen, Carlsbad, CA, USA). RNA quality was verified by formaldehyde agarose gel electrophoresis and quantified with a NanoDrop ND-1000 spectrophotometer. Double-stranded cDNA was synthesized from total RNA samples without rRNA, labeled with cDNA, and hybridized to the New Zealand white rabbit miRNA and mRNA expression microarray v3.0 (8×60 K, Arraystar, Rockville, MD, USA). Following hybridization, the microarrays were washed and scanned with an Agilent Microarray Scanner (Agilent p/n G2565BA). Raw data were extracted as paired files using Agilent Feature Extraction software. Differentially expressed genes were identified using a random variance model, and paired t-tests were conducted to calculate P-values. The thresholds for upregulated and downregulated genes were set at a fold change (FC) > 2.0 and P < 0.05. Hierarchical clustering was performed using clustering software to analyze the expression patterns of miRNAs and mRNAs.

### 2.19 Statistical analysis

All data were expressed as the mean ± standard error of the mean (SEM). Statistical analysis was performed using SPSS 24.0 software. Student’s t-test was used to assess differences between two groups, while one-way ANOVA was employed for multiple group comparisons. p-values < 0.05 were considered statistically significant.

## 3. Result

### 3.1 Establishment of a juvenile rabbit Perthes disease model

We established an avascular necrosis model of the femoral head in juvenile rabbits using femoral neck ligation (Fig 1A). Micro-CT scans were performed on days 0, 14, and 28 to assess the progression of femoral head necrosis (Fig 1B and 1D). The results indicated that necrosis progressively worsened with longer ligation periods. Comparing the femoral heads at days 0, 14, and 28, we observed that the femoral heads at day 28 were larger, flatter, and had a paler overall color compared to those at days 0 and 14 (Fig 1C). After Western blot analysis, we observed a decrease in osteogenic markers such as Runt-related transcription factor (RUNX), Osteopontin (OPN), and Osteocalcin (OC), and an increase in adipogenic markers such as Peroxisome Proliferator-Activated Receptor Alpha (PPARα) (Fig 1E). Concurrently, the expression of B-cell lymphoma-2 (Bcl-2) was downregulated, while Cleaved Caspase-3 (Cleaved CASP-3) and Bcl-2-associated X protein (Bax) were upregulated (Fig 1F).

### 3.2 The expression of miR-223-5p is downregulated during the establishment of the juvenile rabbit Perthes disease model and decreased in BMSCs under hypoxic conditions

Research indicates that a hypoxic environment is generated in the region of femoral head apoptosis, and transfection of mesenchymal stem cells alone is not sufficient to effectively repair the necrotic femoral head [27, 28]. Studies have shown that microRNAs (miRNAs) can regulate gene expression through various mechanisms, such as binding to the 3’ untranslated regions (UTRs) of target mRNAs to inhibit translation or promote degradation, and play significant roles in the regulation of apoptosis [11, 29]. To investigate miRNA changes in the femoral head under hypoxic conditions, we ligated the femoral head and maintained it for 28 days, followed by microarray analysis to obtain miRNA profiles under normal or hypoxic conditions.

**Fig 2:**
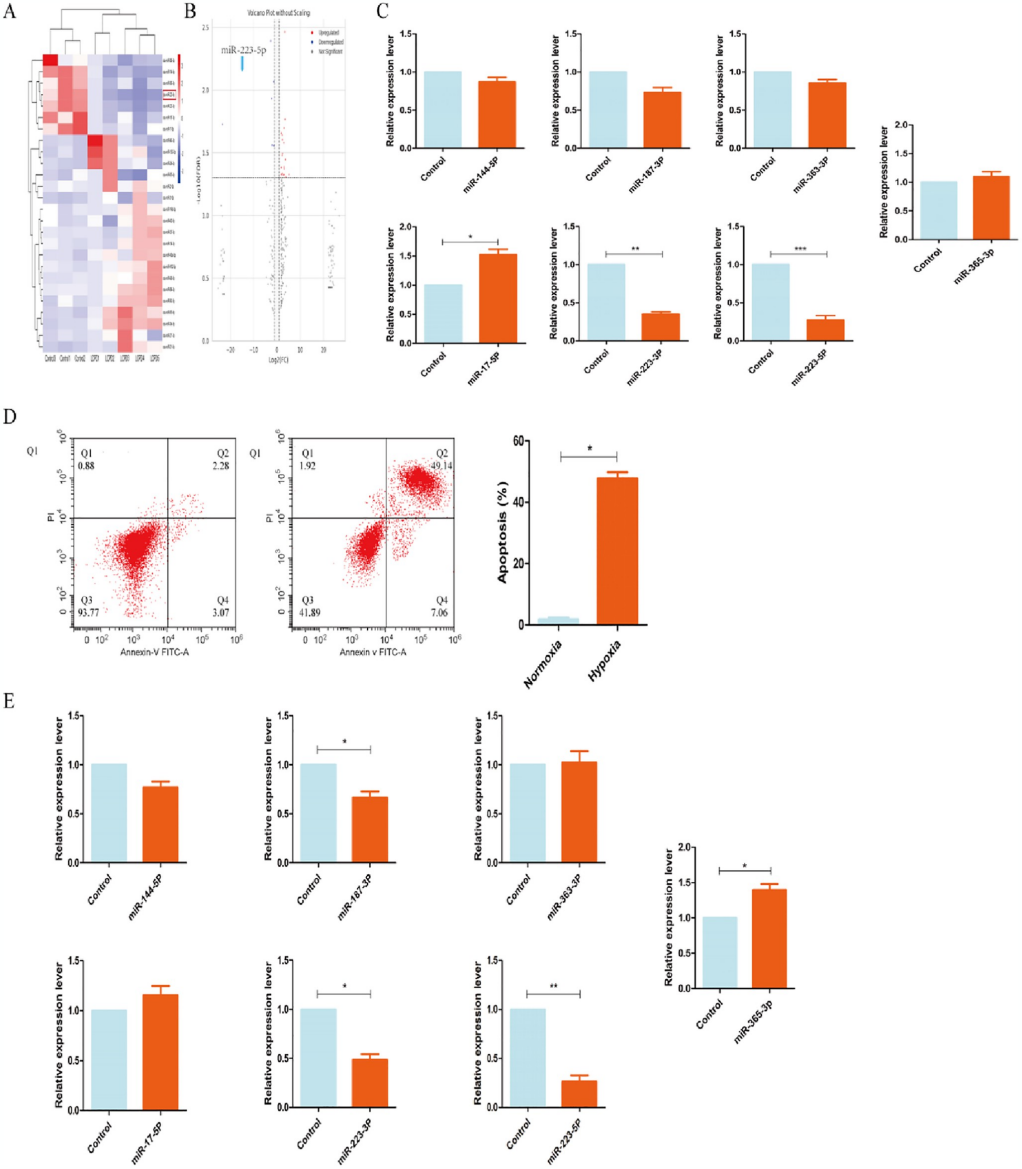
The expression of miR-223-5p is downregulated in the rabbit femoral neck cerclage model and hypoxia BMSCs. Differentially expressed genes were identified based on sequencing results, and PCR was performed on necrotic femoral heads of juvenile rabbits to validate the most significantly differentially expressed genes. Additionally, PCR was conducted on hypoxia-treated BMSCs to identify the most significantly differentially expressed genes based on sequencing data. The target gene was ultimately determined by integrating the results from both analyses. **(A)** Cluster analysis of miRNAs (Control = 3, Model = 5). **(B)** Volcano plot of miRNA expression profiles (Control = 3, Model = 5); fold change (FC) is shown on a log2 scale. **(C)** Expression levels of miR-17-5p, miR-363-3p, miR-223-5p, miR-144-5p, miR-223-3p, and miR-223-5p detected by qPCR between the control group and necrotic femoral heads. **(D)** Apoptosis detected by Annexin V/PI staining. **(E)** Expression levels of miR-223-5p verified by qPCR between the normoxia group and hypoxia group in BMSCs. Data are shown as the means ± sd. *p<0.05, **p<0.01, ***p<0.01 (n = 3).

Our results indicated that 19 miRNAs were upregulated and 7 miRNAs were downregulated under hypoxic conditions, with 7 specific miRNAs being downregulated by more than twofold after hypoxia, as depicted in the heatmap (Fig 2A and 2B). The downregulated miRNAs included miR-363-3p, miR-223-5p, miR-144-5p, miR-223-3p, and miR-365-3p. These miRNAs are known to regulate gene expression through various mechanisms and have been implicated in the regulation of apoptosis. qPCR validation of normal and necrotic femoral heads revealed that miR-223-5p exhibited the most significant downregulation (Fig 2C). To further verify the changes in miRNA expression in vitro, we subjected BMSCs to hypoxic conditions and found that the expression of miR-223-5p was significantly downregulated in the hypoxia group compared to the normoxia group (Fig 2E).

### 3.3 MiR-223-5p can inhibit hypoxia-induced apoptosis of BMSCs and activate the β-catenin signaling pathway in vitro

**Fig 3:**
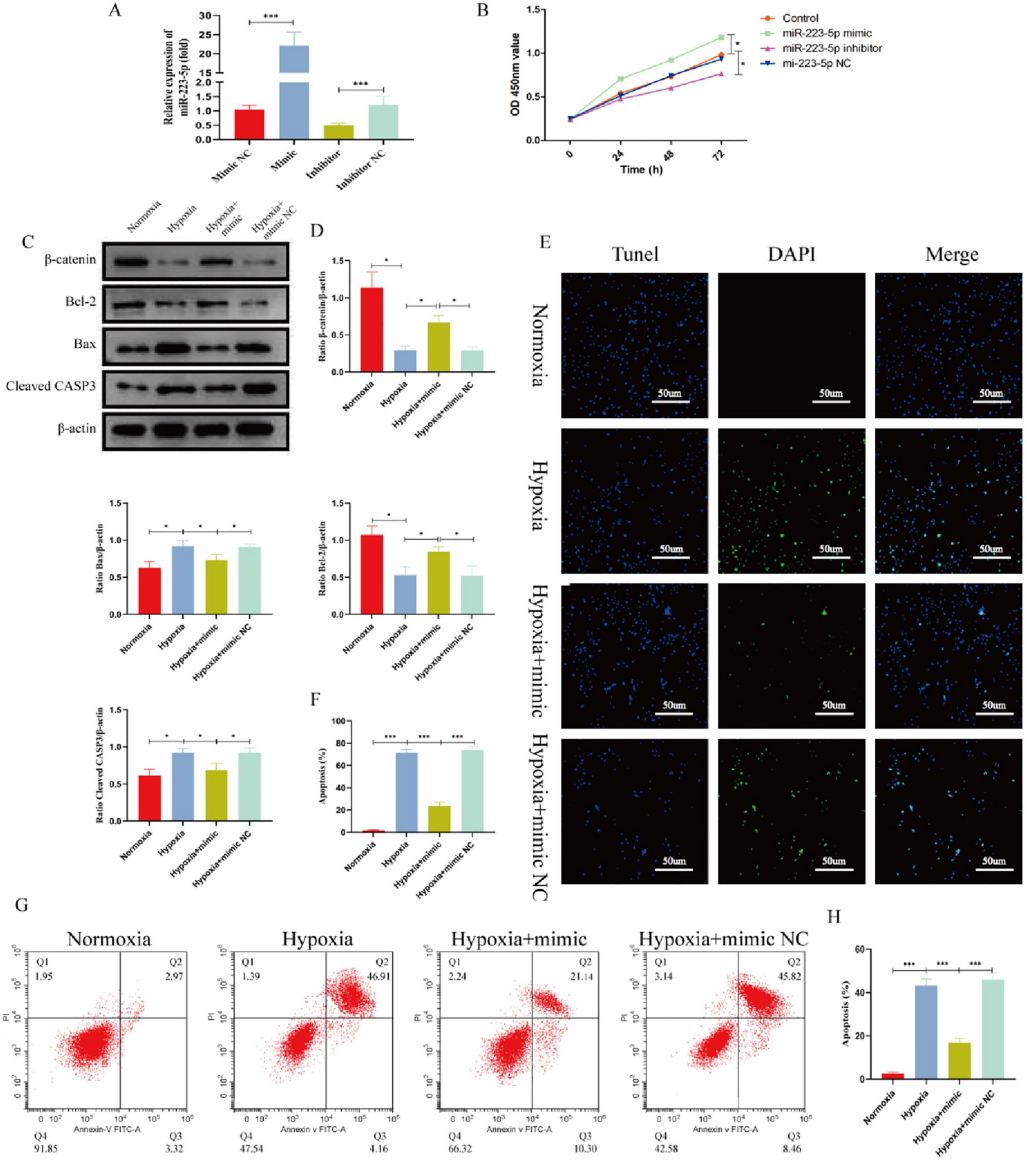
miRNA-223-5p inhibits hypoxia-induced apoptosis of BMSCs and activate the β-catenin signaling pathway. After transfecting miRNA-223-5p into BMSCs, the cells were cultured under hypoxic conditions. Apoptosis in BMSCs was evaluated using Western blot (WB), TUNEL staining, and flow cytometry. Additionally, the activation of the Wnt/β-catenin signaling pathway was assessed. **(A)** The expression of miRNA-223-5p was detected by qPCR. **(B)** The viability of BMSCs transfected with miRNA was measured using the CCK-8 assay. **(C,D)** The expression levels of β-catenin, Bcl-2, Bax, and Cleaved CASP-3 were analyzed by Western blotting. **(E,F)** TUNEL (blue) and DAPI (green) double immunofluorescence staining were used to detect apoptosis. **(G,H)** Apoptosis was detected using Annexin V/PI staining. Data are shown as the means ± SD. *p<0.05, **p<0.01, ***p<0.01 (n = 3).

To further investigate the effects of miR-223-5p on hypoxia-induced apoptosis of BMSCs, we transfected BMSCs with miR-223-5p mimics, mimic NC, inhibitor, and inhibitor NC using Lipofectamine 3000. After transfecting miR-223-5p into BMSCs, the expression of miR-223-5p increased significantly (Fig 3A). As shown in Fig 3B, oligo transfection significantly impacted BMSC proliferation, as detected by the CCK-8 assay. Then, BMSCs were exposed to hypoxia for 48 hours. The results showed that under hypoxia, β-catenin and Bcl-2 expression levels were downregulated, while Bax and Cleaved CASP-3 expression levels were upregulated (Fig 3C and 3D), with the BMSC apoptotic rate exceeding 70% (Fig 3E and 3F). However, overexpression of miR-223-5p reversed these effects, significantly reducing the apoptotic rate of BMSCs and promoting their survival under hypoxia while inhibiting the β-catenin signaling pathway (Fig 3G and 3H). Notably, β-catenin expression was clearly upregulated by miR-223-5p overexpression. These findings suggest that miR-223-5p inhibits hypoxia-induced apoptosis of BMSCs and activate the β-catenin signaling pathway.

### 3.4 CHAC2 mRNAs are direct targets of miR-223-5p

**Fig 4:**
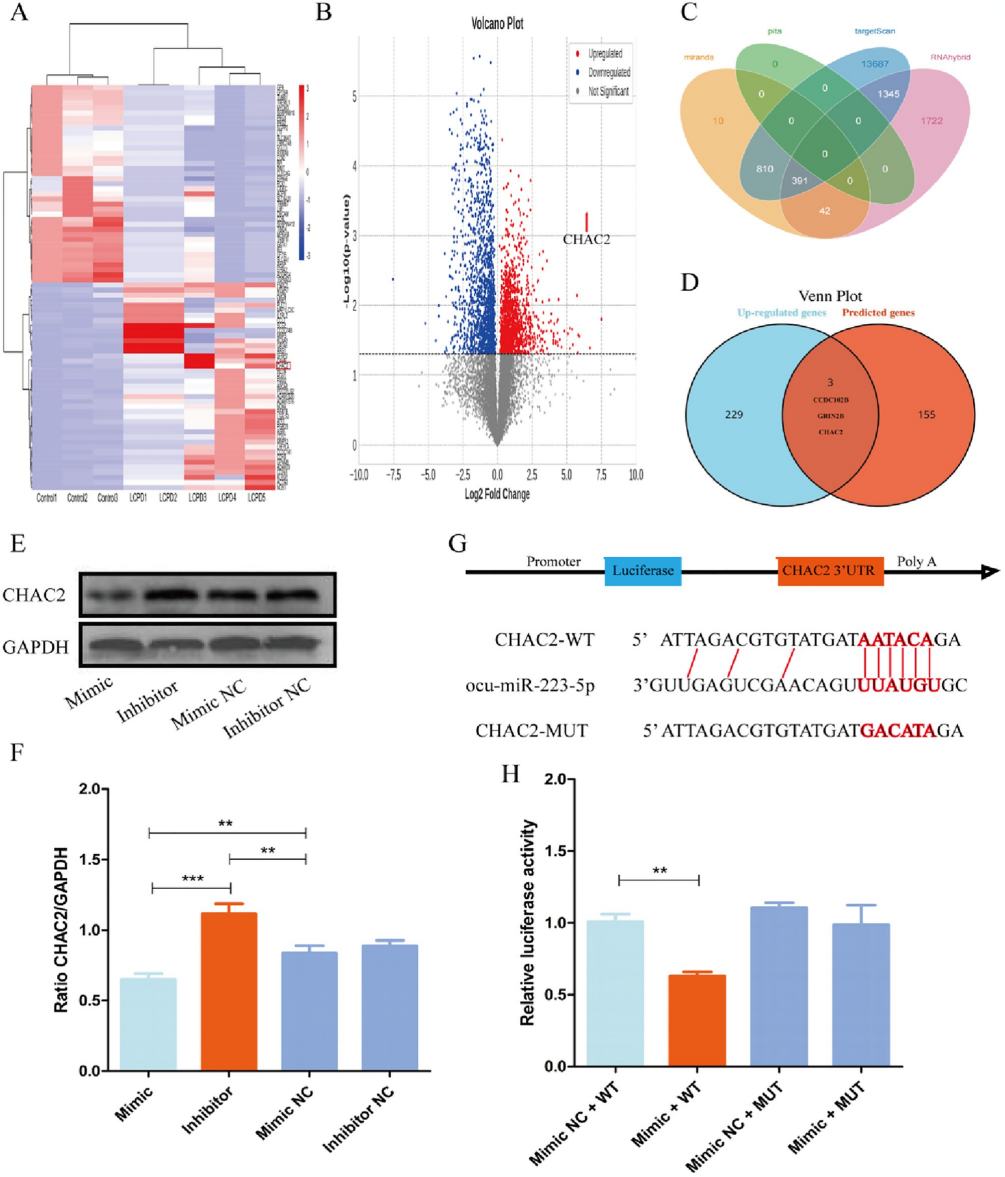
CHAC2 directly interacts with miR-223-5p. Differentially expressed genes were identified in mRNA, and potential target genes were predicted using online databases, including TargetScan, RNAhybrid, PITA, and miRanda. The direct interaction between the miRNA and the target gene was subsequently validated using a luciferase reporter gene assay. **(A)** Cluster analysis of mRNAs (Control = 3, Model = 5). **(B)** Volcano plot of mRNA expression profile (Control = 3, Model = 5); fold change (FC), with the base of logFC set at 2. **(C)** Bioinformatics analysis predicted potential miRNA targets that interact with mRNAs. **(D)** Upregulated genes and predicted genes were used to screen candidate mRNAs. **(E, F)** The protein expression of CHAC2 was assessed in BMSC cells co-transfected with miR-223-5p. **(G)** The ocu-miR-223-5p seed sequence and the putative binding sequences in the 3’UTR of CHAC2; both wild-type and mutant-type sequences were inserted into constructs. **(H)** HEK293 cells were co-transfected with oligos and constructs. Luciferase activities were examined, and the firefly luciferase activities of each sample were normalized to Renilla luciferase activities. Data are shown as the means ± SD. *p<0.05, **p<0.01, ***p<0.01 (n = 3).

To further explore the interaction between miRNA and mRNA, we ligated the femoral head and maintained it for 28 days. We then used microarray analysis to obtain RNA profiles of the femoral head under normal or hypoxic conditions. Our results indicated that 6218 mRNAs were upregulated and 5872 mRNAs were downregulated under hypoxic conditions, with 232 potential miRNAs being upregulated by more than 2-fold after hypoxia, as shown in the heatmap (Fig 4A and 4B). To investigate the potential mechanism of miR-223-5p in rabbit BMSCs, we speculated the promising targets of miR-223-5p using miRanda, PITA, TargetScan, and RNAhybrid. A total of 391 genes were found to have common intersections (Fig 4C). By intersecting the database-predicted genes with the upregulated genes identified through microarray analysis, we identified three genes: CCDC102B, GRIN2B, and CHAC2 (Fig 4D). Current research suggests that CCDC102B is primarily involved in the development of myopic macular degeneration and tumorigenesis [30, 31], while GRIN2B is mainly involved in neurotransmitter transmission [32]. Given that CHAC2 acts as a primary enzyme for GSH degradation [33] and is associated with apoptosis, we considered CHAC2 as a potential mediator through which miR-223-5p influences the apoptosis of BMSCs under hypoxic conditions. Furthermore, miR-223-5p can suppress the expression of CHAC2 at the protein level. Conversely, inhibition of miR-223-5p leads to an upregulation of CHAC2 expression (Fig 4E and 4F). Additionally, dual luciferase assays demonstrated that miR-223-5p reduced the luciferase activity of wild-type CHAC2 constructs compared to mutant groups. The positive control confirmed the validity of the method (Fig 4G and 4H).

### 3.5 MiR-223-5p inhibits hypoxia-induced apoptosis of BMSCs by regulating CHAC2 and activate the β-catenin signaling pathway

**Fig 5:**
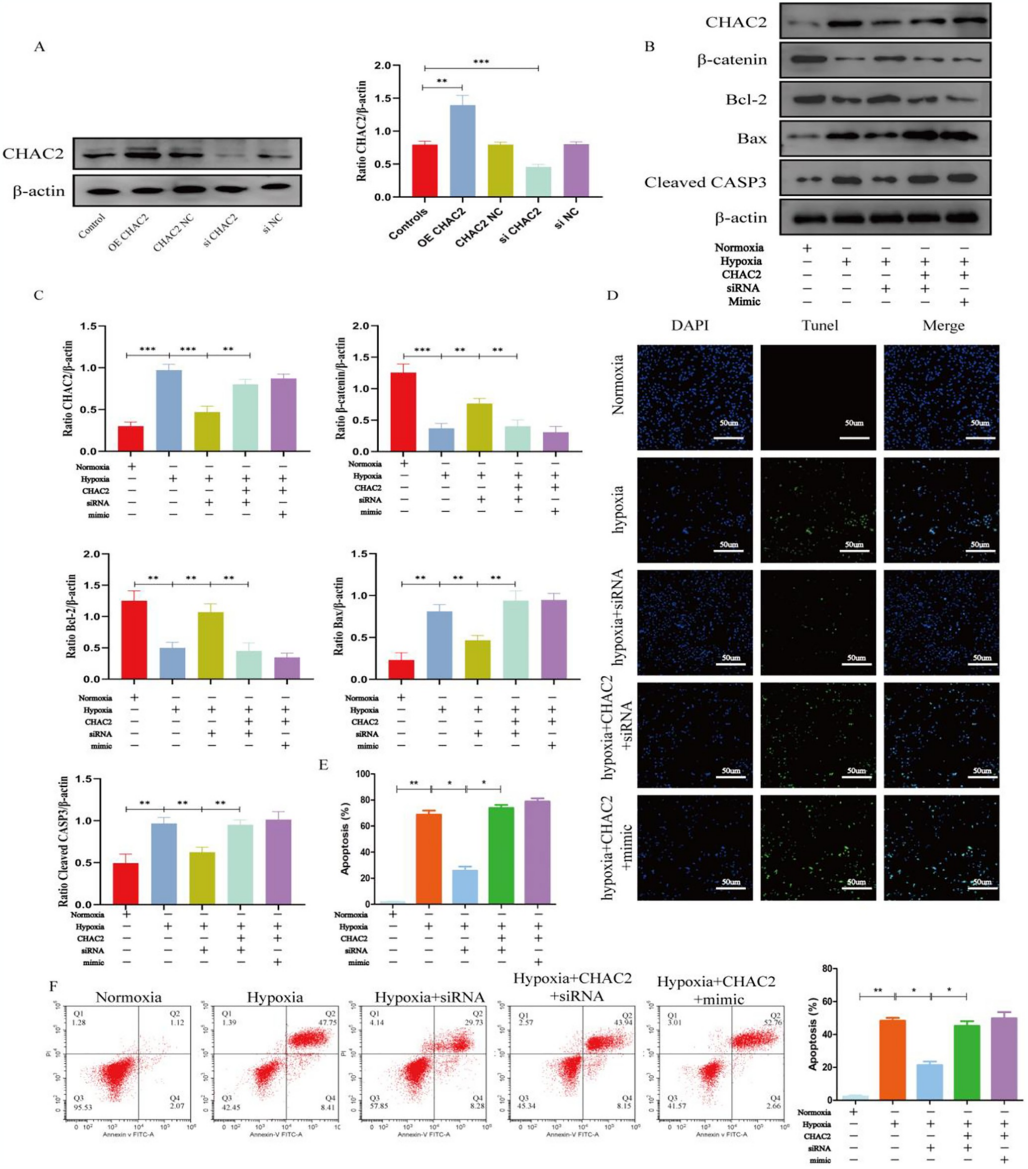
Overexpression of miR-223-5p rescued the effects of CHAC2. BMSCs overexpressing CHAC2 were constructed, and miRNA-223-5p was transfected into BMSCs. The cells were then cultured under hypoxic conditions. Apoptosis in BMSCs was evaluated using Western blot (WB), TUNEL staining, and flow cytometry. Additionally, the activation of the Wnt/β-catenin signaling pathway was assessed. **(A, B)** The expression levels of CHAC2 were analyzed by Western blotting. **(B, C)** The expression levels of β-catenin, Bcl-2, Bax, and Cleaved CASP-3 were analyzed by Western blotting(siRNA. **(D, E)** TUNEL (blue) and DAPI (green) double immunofluorescence staining were used to detect apoptosis. **(F)** Apoptosis was detected using Annexin V/PI staining. Data are shown as the means ± SD. *p<0.05, **p<0.01, ***p<0.01 (n = 3).

We first constructed a lentivirus for CHAC2 overexpression in BMSCs and transfected BMSCs with CHAC2 siRNA. It was found that CHAC2 levels in BMSCs exhibited significant changes (Fig 5A). We next determined whether miR-223-5p inhibited hypoxia-induced apoptosis of BMSCs by regulating CHAC2. We inhibited CHAC2 by downregulating CHAC2 expression using gene-specific small interfering (si)RNAs, and then subjected BMSCs to hypoxia for 48 h. After silencing with siRNA, it was observed that, compared to the hypoxia group, β-catenin and Bcl-2 gene expression was upregulated, while CHAC2, Bax and Cleaved CASP-3 gene expression was downregulated (Fig 5B and 5C). Simultaneously, the apoptosis rate of BMSCs decreased. In the subsequent rescue experiment, an increase in CHAC2 gene expression led to a rise in the apoptosis rate of BMSCs, suggesting that the CHAC2 gene can induce apoptosis in BMSCs (Fig 5D–5F). Furthermore, co-transfection of BMSCs overexpressing the CHAC2 gene with a mimic led to an even higher apoptosis rate compared to BMSCs transfected with the mimic alone (Fig 3D–3H). This indicates that miR-223-5p can regulate BMSC apoptosis through CHAC2 and activate the β-catenin signaling pathway.

### 3.6 Repair effects of transplantation of miR-223-5p-overexpressed BMSCs into the Perthes model

**Fig 6:**
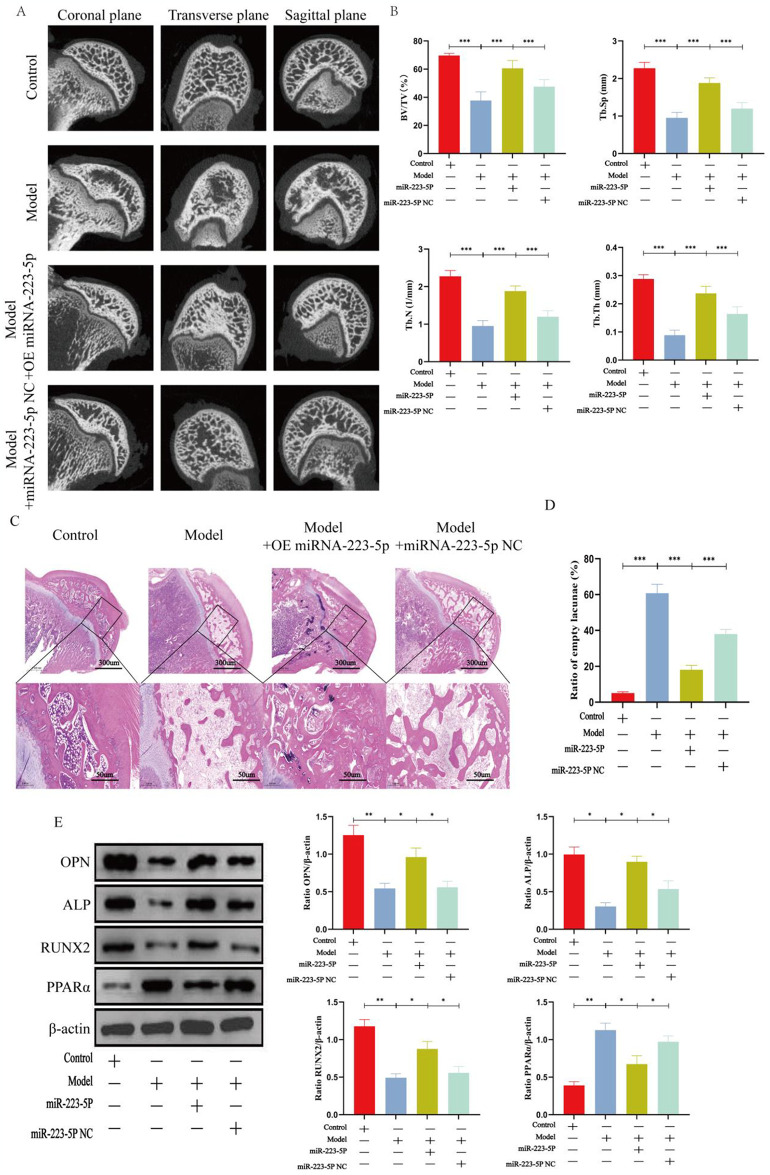
Transplantation of miR-223-5p-overexpressed BMSCs enhanced osteogenesis in vivo. After transfecting miRNA-223-5p into BMSCs, the modified BMSCs were locally injected into the necrotic region of the femoral head. The repair of the femoral head was assessed using micro-CT, hematoxylin and eosin (HE) staining, and the evaluation of bone metabolism markers. **(A)** Micro-CT scanning images of the femoral head in the coronal, transverse, and sagittal planes were reconstructed for the four groups. **(B)** Evaluation of trabecular parameters, including BV/TV, Tb.N, Tb.Th, and Tb.Sp, was performed based on micro-CT scanning. **(C-D)** Coronal plane sections stained with H&E were used to observe the trabecular bone structure of the femoral heads, and the ratios of empty lacunae were measured. **(E)** The levels of osteogenic markers (Runx2, OPN, PPAR, and CEBP-α) were detected by Western blotting. Data are shown as the means ± sd. *p<0.05, **p<0.01, ***p<0.01 (n = 3).

The effect of miR-223-5p-overexpressed BMSC transplantation was analyzed in the juvenile rabbit Perthes disease model. First, the juvenile rabbit Perthes disease model was successfully generated, as shown in Fig 1A. Micro-CT scanning results showed that the NC group exhibited only slight improvement compared to the model group. By contrast, miR-223-5p-overexpressed BMSC transplantation significantly attenuated the pathological changes (Fig 6A). Furthermore, femoral neck ligation seriously deteriorated trabecular parameters, such as BV/TV, Tb.N, Tb.Th, and Tb.Sp. However, miR-223-5p-overexpressed BMSC transplantation significantly improved these parameters, while they were only slightly restored in the NC group (Fig 6B). When examined histologically, miR-223-5p-overexpressed BMSC transplantation clearly enhanced osteogenesis in the juvenile rabbit Perthes disease model. Bone histomorphometry results showed more trabecular bone structure and fewer empty lacunae in the femoral head of the miR-223-5p-overexpressed group compared to the model or NC groups (Fig 6C and 6D). WB showed that Runx2, OCN, and OC were downregulated, and PPARα was upregulated in the model group. The expression of Runx2, OCN, and OC was significantly restored by miR-223-5p-overexpressed BMSC transplantation (Fig 6F).

## 4. Discussion

Perthes’ disease is a self-limiting condition in children caused by an interrupted blood supply to the femoral epiphysis, leading to necrosis [34]. Due to the difficulty in obtaining clinical specimens and the ethical challenges of conducting experimental studies in children, most research on LCPD relies on animal models. Unlike adult femoral head necrosis, the immature hip joint in children demonstrates greater plasticity and remodeling potential, allowing for the possibility of the femoral head maintaining its position within the acetabulum and restoring its sphericity. The development of in vivo models of femoral head necrosis in immature hips and the exploration of therapeutic interventions, such as bone marrow stem cells (BMSCs), are essential for advancing clinical treatments for LCPD. Our study contributes to this effort by establishing a novel rabbit model of Perthes disease and investigating the potential of miR-223-5p to enhance BMSC survival under hypoxic conditions, thereby providing new insights into improving therapeutic strategies.

In young animals and children, the proximal femoral growth plate’s trophoblastic vascular region relies on external vasculature due to the lack of internal blood vessels [35]. This dependency makes the femoral head more prone to ischemic injury. Researchers have developed various animal models to study this disease by disrupting the blood flow to the femoral epiphysis. Martínez-Álvarez et al. induced ischemia in the proximal femur of lambs to create a Perthes’ disease model [36]. Norman and Chen caused femoral head necrosis in rats through vascular deprivation [37, 38]. Wang et al. developed a juvenile rabbit model by disrupting the femoral round ligament [8]. Currently, many scholars predominantly use a piglet model of ischemic necrosis, which is established by placing a non-absorbable ligature tightly around the femoral neck to cut off the blood supply to the capital femoral epiphysis [9, 39]. Due to the limitations of space, large-scale rearing of piglets is not feasible; therefore, we established a Perthes disease model using rabbits. Similar to the piglet model of Perthes disease, we cut the ligament of the femoral head and tightly ligated the base of the femoral neck with non-absorbable sutures. This method successfully created a rabbit model of Perthes disease. This model not only replicates the pathological features observed in larger animal models but also introduces a novel approach that enhances feasibility and applicability in experimental research.

Numerous research groups are currently investigating the use of MSCs for treating osteonecrosis of the femoral head, utilizing various procedures, time points, and dosages [8, 9, 40]. The rationale for cell therapy in this context is supported by the reduced numbers and impaired function of MSCs in the necrotic area [41]. MSCs can differentiate into bone, cartilage, or fat, providing osteogenic precursors and promoting healing through angiogenesis and fibrosis inhibition [42]. However, the hypoxic environment of the osteonecrotic area challenges BMSC survival, posing a major obstacle for successful transplantation [27, 43]. It has been found that numerous miRNAs are abnormally expressed in cells under hypoxic conditions and play crucial roles in regulating cell function [24, 44].

In the present study, we identified a key role of miR-223-5p in reducing hypoxia-induced apoptosis of BMSCs. Specifically, we found that miR-223-5p can spong with CHAC2 mRNA to inhibiting hypoxia-induced apoptosis of BMSCs by activing wnt/β-catenin. Additionally, we found that miR-223-5p overexpression effectively reduced hypoxia-induced apoptosis of BMSCs, improving the transplantation effect of BMSCs in treating early LCPD. In this study, we demonstrated the crucial role of miR-223-5p in mitigating hypoxia-induced apoptosis in BMSCs. Our findings reveal that miR-223-5p interacts with CHAC2 mRNA to inhibit hypoxia-induced apoptosis by activating the Wnt/β-catenin pathway. Furthermore, overexpression of miR-223-5p significantly enhanced the transplantation efficacy of BMSCs in treating Perthes disease.

In our study, the overexpression of miR-223-5p was validated by the significant downregulation of Bax and Cleaved CASP-3 expression, accompanied by the upregulation of Bcl-2 and β-catenin in BMSCs. These molecular changes indicate the protective role of miR-223-5p in enhancing cell survival under hypoxic conditions. Several studies have investigated the functions of miR-223-5p. It was discovered that miR-223-5p reduces monocyte infiltration by targeting CCR2 activation and promotes angiogenesis, thereby alleviating myocardial ischemia-reperfusion injury [18]. Moreover, research has suggested that miR-223-5p suppresses the progression of nasopharyngeal carcinoma by targeting DCLK1 [44]. MicroRNA-223 has been shown to promote osteoblast differentiation of MC3T3-E1 cells by targeting histone deacetylase 2 [17]. However, its role in regulating BMSC survival under hypoxia has not been previously described. Our study is the first to reveal this novel function of miR-223-5p, thereby filling a significant gap in the understanding of miRNA-mediated regulation of BMSCs in hypoxic conditions.

By sequencing the femoral heads of young rabbits, we identified seven miRNAs with p-values less than 0.05 and logFC greater than 2. We further validated these findings through PCR analysis of both the femoral heads and the BMSC cells used in our study. Among these, miR-223-5p showed the most significant change. Notably, the regulatory role of miR-223-5p in BMSC cells remains unexplored. Our study revealed that miR-223-5p markedly reduces hypoxia-induced apoptosis in BMSC cells. Additionally, luciferase reporter assays demonstrated that miR-223-5p directly interacts with the CHAC2 gene, inhibiting its function and consequently reducing cell apoptosis. The highly conserved nature of miRNA sequences across different species results in minimal sequence variations, allowing miRNA studies to be broadly applicable across multiple species. Our analysis using the human TargetScan database revealed a high probability of miR-223-5p binding to the CHAC2 sequence in humans, suggesting that our findings may be relevant to human cellular studies. However, further validation in BMSCs is necessary to confirm this relevance. Given the inherent challenges of conducting experiments on human cells, we opted to use young rabbits in this study to maintain consistency between cellular and animal models. This novel finding provides a new molecular target for enhancing BMSC survival under hypoxic conditions, which is crucial for the success of cell-based therapies in osteonecrosis. Our approach of targeting miR-223-5p represents a significant advancement over previous studies that have not addressed the challenge of hypoxia-induced apoptosis in BMSCs.

CHAC2, a member of the CHAC family, functions as a glutathione-degrading enzyme and is crucial in maintaining the pluripotency of human embryonic stem cells (hESCs). It is highly expressed in undifferentiated hESCs and induced pluripotent stem cells, highlighting its role in stem cell biology [24]. Current research on CHAC2 predominantly focuses on tumor biology, with limited exploration of its involvement in bone-related studies. However, the role of CHAC2 in apoptosis has garnered increasing attention. For example, in a study of hydrogen peroxide-induced apoptosis in skin cells, isoviolanthin was shown to potentially interact with CHAC2, thereby mitigating cell apoptosis [45]. Similarly, studies in gastric and colorectal cancers have demonstrated that CHAC2 overexpression significantly enhances tumor cell apoptosis, contributing to improved patient survival outcomes [46, 47]. In our study, we found that siCHAC2 inhibits hypoxia-induced apoptosis in BMSCs, while CHAC2 overexpression under hypoxic conditions promotes cell apoptosis and increases the levels of Bax and Bcl-2. Furthermore, miR-223-5p can directly bind to CHAC2, thereby reducing hypoxia-induced apoptosis in BMSCs.

There is a substantial body of literature reporting the effects of hypoxia on the Wnt/β-catenin signaling pathway. Hypoxia can promote the Wnt/β-catenin signaling pathway through several mechanisms. Stabilized hypoxia-inducible factors (HIFs) under hypoxic conditions can bind to β-catenin, enhancing its nuclear activity and activating the pathway. This interaction boosts β-catenin’s interaction with TCF/LEF transcription factors, promoting Wnt target gene expression [48]. HIFs also upregulate genes like Wnt3a, enhancing signaling by regulating Wnt ligands and receptors [49]. Additionally, the reduced activity of oxygen-dependent hydroxylases under hypoxia decreases β-catenin degradation, increasing its stability and activity, leading to its nuclear translocation and pathway activation [50]. Conversely, hypoxia can inhibit the Wnt/β-catenin signaling pathway through various mechanisms. Increased levels of ROS under hypoxia can damage key Wnt signaling molecules, such as Frizzled receptors and β-catenin, disrupting signal transduction [48, 51]. Metabolic stress and reduced ATP production activate AMPK, which promotes β-catenin degradation [52]. Lastly, prolonged hypoxia may activate VHL-mediated degradation of β-catenin, further inhibiting the pathway [51]. In our study, we found that complete hypoxia conditions inhibit the expression of the Wnt/β-catenin signaling pathway. However, upon transfection with miR-223-5p, the Wnt/β-catenin signaling pathway is partially reactivated. Similarly, in rescue experiments, we observed that silencing CHAC2 also partially reactivates the Wnt/β-catenin signaling pathway. There is a direct interaction mechanism between miR-223-5p and CHAC2, and both positive and negative regulation indicate alterations in the Wnt signaling pathway. Although we did not conduct Wnt signaling pathway blockade experiments, based on previous studies on Wnt and hypoxia, we believe that the Wnt signaling pathway is one of the pathways through which miR-223-5p and CHAC2 regulate apoptosis of BMSCs under hypoxic conditions. By uncovering this novel regulatory axis, our study opens new avenues for future research to explore therapeutic strategies that target the miR-223-5p/CHAC2/Wnt/β-catenin pathway to enhance BMSC survival and function under hypoxic conditions.

Previous studies have demonstrated that the transplantation of BMSCs alone in early-stage femoral head necrosis models yields unsatisfactory outcomes [10]. This is primarily due to the high rate of BMSC apoptosis in the hypoxic microenvironment of the necrotic region, which significantly limits the therapeutic efficacy of BMSC transplantation for early-stage femoral head necrosis [27]. This limitation significantly hampers the therapeutic efficacy of BMSC transplantation for early-stage femoral head necrosis. Consistent with these findings, our animal study also observed suboptimal femoral head repair following the implantation of BMSCs alone. Our study advances the current understanding by demonstrating that overexpression of miR-223-5p in BMSCs can inhibit hypoxia-induced apoptosis, thereby enhancing cell survival in the harsh microenvironment of the necrotic region. We evaluated the in vivo reparative effects of miR-223-5p by transplanting BMSCs overexpressing miR-223-5p in an early-stage LCPD model in young rabbits. The results showed that miR-223-5p-mediated inhibition of hypoxia-induced apoptosis in BMSCs significantly enhanced the therapeutic efficacy of BMSC transplantation. These findings provide new insights into improving BMSC-based therapies for osteonecrosis by targeting miR-223-5p to overcome the challenge of hypoxia-induced apoptosis.

## 5. Conclusion

**Fig 7:**
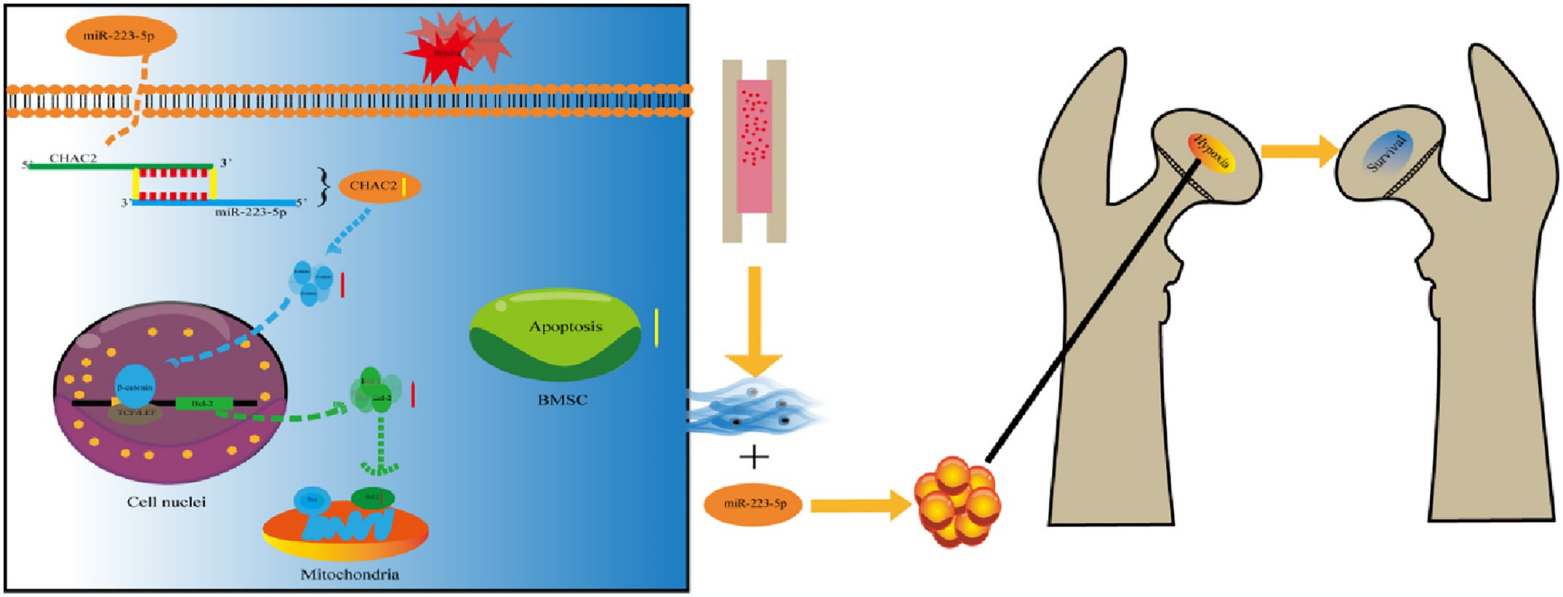
A schematic illustration depicts how miR-223-5p regulates CHAC2 to inhibit hypoxia-induced apoptosis in BMSCs.

In summary, we found that miR-223-5p and CHAC2 play crucial roles in modulating the Wnt/β-catenin signaling pathway under hypoxia. Complete hypoxia conditions inhibit this pathway in BMSCs, likely due to increased ROS causing oxidative stress and cell damage. However, transfection with miR-223-5p or silencing CHAC2 partially reactivates the Wnt/β-catenin pathway, mitigating the inhibitory effects of hypoxia. miR-223-5p acts as a sponge for CHAC2 mRNA, reducing hypoxia-induced apoptosis by activating the Wnt/β-catenin pathway. We also systematically evaluated the therapeutic effect of miR-223-5p-overexpressing BMSC transplantation in Perthes disease, providing an effective molecular strategy and a novel target for improving BMSC transplantation efficacy (Fig 7).

## Supporting information

S1 Raw images (PDF)
