# Supplementary material for: MiRNA-223-5p inhibits hypoxia-induced apoptosis of BMSCs and promotes repair in Legg-Calvé-Perthes disease by targeting CHAC2 and activating the Wnt/β-catenin signaling pathway
Source: PLoS One. 2025 Jan 24;20(1):e0315230. doi: 10.1371/journal.pone.0315230 (PMC11761568; doi:10.1371/journal.pone.0315230)
Supplement: S1 Raw images — (PDF) [file pone.0315230.s001.pdf]

miRNA-223-5p Inhibits Hypoxia-induced Apoptosis of BMSCs and Promotes Repair in a Rabbit Model of Legg-Calvé-Perthes Disease by Targeting CHAC2 and Activating the Wnt/β-Catenin Signaling Pathway

Supplementary Figure 1: The uncropped images of the original western blots.

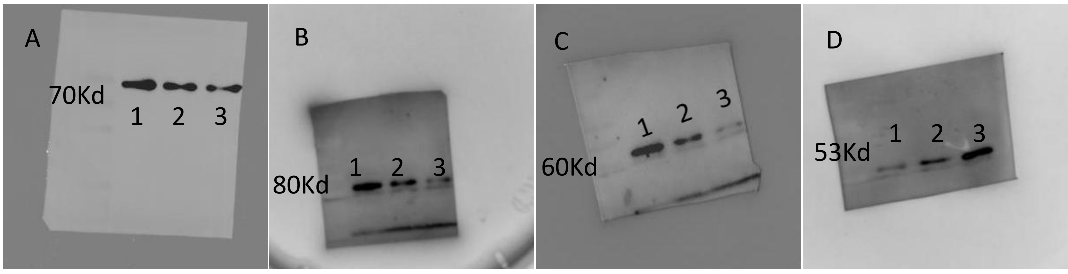

Supplementary figure from Figure 1E in the main manuscript: The expression of the protein. (A) OPN protein band detected by Western blots; (B) ALP protein band detected by Western blots; (C) RUNX2 protein band detected by Western blots; (D) PPARα protein band detected by Western blots;

Note:1:Control; 2:Day 14; 3:Day 28

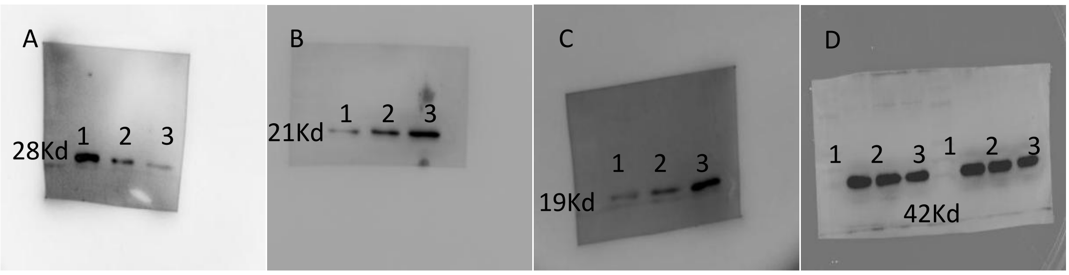

Supplementary figure from Figure 1F in the main manuscript: The expression of the protein. (A) Bcl-2 protein band detected by Western blots; (B) Bax protein band detected by Western blots; (C) Cleaved Caspase3 protein band detected by Western blots; (D) β-actin protein band detected by Western blots.

Note:1:Control; 2:Day 14; 3:Day 28

Supplementary Figure 2:

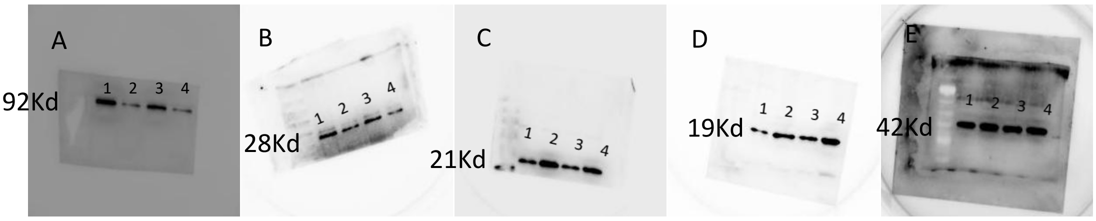

Supplementary figure from Figure 3C in the main manuscript: The expression of the protein. (A)β-catenin protein band detected by Western blots; (B) Bcl-2 protein band detected by Western blots; (C) Bax protein band detected by Western blots; (D) Cleaved Caspase3 protein band detected by Western blots; (E) β-actin protein band detected by Western blots.

Note:1:Normoxia; 2:Hypoxia ; 3:Hypoxia+mimic; 4: Hypoxia+mimicNC

Supplementary Figure 3:

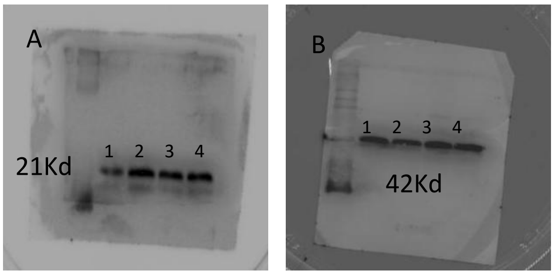

Supplementary figure from Figure 4E in the main manuscript: The expression of the protein. (A) CHAC2 protein band detected by Western blots; (B) GAPDH protein band detected by Western blots.

Note:1:mimic; 2:inhibitor ; 3:mimicNC; 4 inhibitor NC

Supplementary Figure 4:

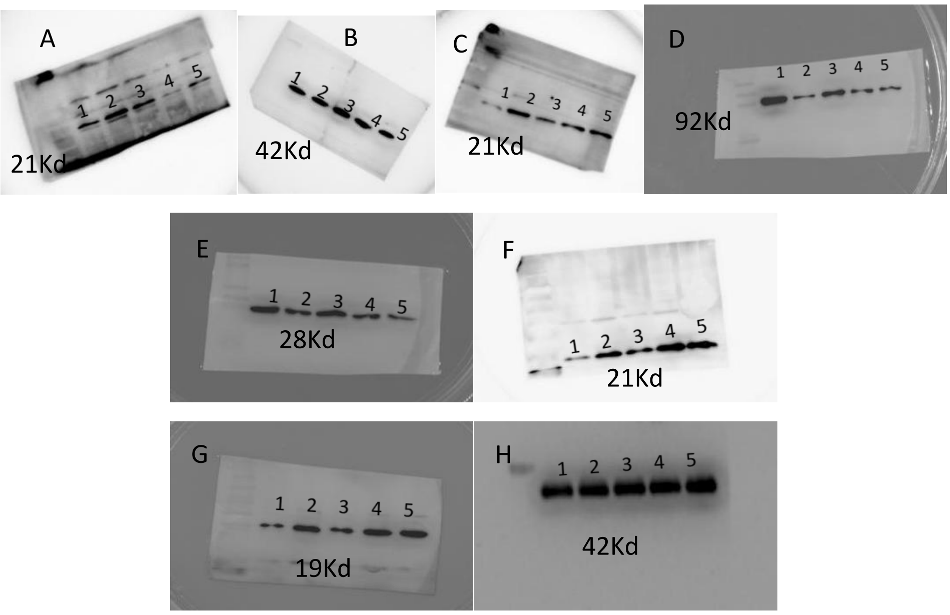

Supplementary figure from Figure 5in the main manuscript: The expression of the protein. (A)CHAC2 protein band detected by Western blots; (B)β-actin protein band detected by Western blots; (C)CHAC2 protein band detected by Western blots; (D)β-catenin protein band detected by Western blots; (E) Bcl-2 protein band detected by Western blots; (F) Bax protein band detected by Western blots; (G) Cleaved Caspase3 protein band detected by Western blots; (H) β-actin protein band detected by Western blots.

(A-B) Note:1:Control; 2:OE CHAC2; 3:CHAC2 NC; 4:siCHAC2; 5:siNC

(C-H) Note:1:Normoxia; 2:Hypoxia ; 3:Hypoxia+siRNA;

4: Hypoxia+siRNA+CHAC2; 5:Hypoxia+mimic+CHAC2

Supplementary Figure 5:

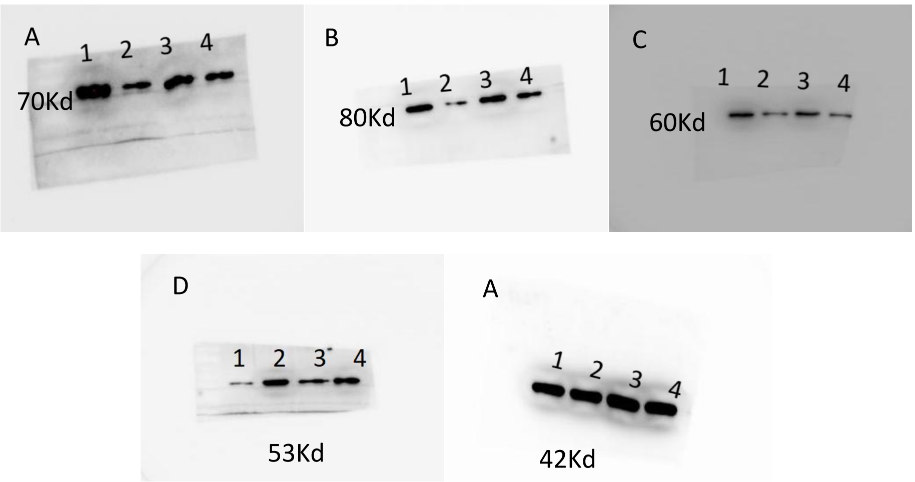

Supplementary figure from Figure 1E in the main manuscript: The expression of the protein. (A) OPN protein band detected by Western blots; (B) ALP protein band detected by Western blots; (C) RUNX2 protein band detected by Western blots; (D) PPARαprotein band detected by Western blots;(E) β-actin protein band detected by Western blots.

Note: 1:Control; 2:Model; 3:Model+mimic; 4:Model+mimic NC

Antigen-antibody complexes were stained with Clarity Western ECL substrate(Bio-Rad) and visualization was performed using an image scanner(ChemiDoc XRS Plus Imaging System, Bio-Rad)
